# Demystifying Dens Invaginatus: Suggested Modification of the Classification based on a Comprehensive Case Series

**DOI:** 10.14744/eej.2021.48303

**Published:** 2022-03-14

**Authors:** Selvakumar KRITIKA, Sweta Surana BHANDARI, Gergely BENYÖCS, Paula Andrea VILLA MACHADO, Nirmala BISHNOI, Felipe Augusto RESTREPO-RESTREPO, Kittappa KARTHIKEYAN, Ida de ATAIDE, Sekar MAHALAXMI

**Affiliations:** From the Department of Conservative Dentistry and Endodontics (S.K. 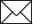 drkritikaselvakumar22@gmail.com, K.K., S.M.), SRM Dental College, Ramapuram, SRM Institute of Science & Technology, Tamil Nadu, India; Private Practitioner (S.S.B.), Smile Dental Clinic, Muscat, Oman; Private Practitioner (G.B.), Precedent Dental Office, Budapest, Hungary; POPCAD Research Group (P.A.V.M., F.A.R.R.), Laboratory of Immunodetection and Bioanalysis, University of Antioquia, Faculty of Dentistry, Medellín, Colombia; Department of Conservative Dentistry and Endodontics (N.B.), Vyas Dental College and Hospital, Rajasthan, India; Department of Conservative Dentistry and Endodontics (I.D.A.), Goa Dental College and Hospital, Goa, India

**Keywords:** Anatomy, CBCT, classification, dens invaginatus, maxillary lateral incisor, periapical lesion

## Abstract

Dens invaginatus (DI) is one of the most common developmental anomalies observed in maxillary lateral incisors. An early diagnosis requires thorough clinical knowledge, advanced radiographic evaluation as well as the use of cone beam computed tomography (CBCT) and dental operating microscope (DOM), dictates the successful endodontic management of such teeth. A total of 7 cases with (DI) in maxillary lateral incisors were collected from dental practitioners worldwide, analysed and reported in the present case series. Our aim was to analyse and understand the various morphological patterns of DI in maxillary lateral incisors with their varied treatment protocols employed worldwide. This article illustrates the aberrant morphological patterns and the diverse treatment protocols followed by the clinicians worldwide. The use of biomaterials enhances post-operative healing. Further, a modification in the existing classification has been proposed in this report which would enable the clinicians to easily diagnose, categorise and effectively manage DI. The different treatment protocols employed for the management of DI has been discussed and the use of CBCT and DOM in identifying and managing the anatomical variation of DI were emphasised.

**HIGHLIGHTS**

• The altered morphological patterns of dens invaginatus and the varied treatment protocols followed by the clinicians worldwide has been discussed.
• The categorization of Oheler’s DI Type II into 3 types will enable the clinicians to better understand and manage the treatment protocol such cases.
• The use of cone beam computed tomography (CBCT) and dental operative microscope (DOM) in identifying and managing the anatomical variation of invaginatus were emphasised.

## INTRODUCTION

*Dens invaginatus* (DI) is a common dental developmental anomaly in the maxillary lateral incisors with a frequent bilateral prevalence (1). The term “*Dens invaginatus*” was coined by Hallet in 1953 who also introduced the first classification. This classification was later rearranged by Oehlers in 1957 (2) [as stated by Alani et al. (3)] (Table 1). Literature shows a prevalence ranging from 0.04% to 10% (3, 4). These variations are mainly attributed to the methodological differences in cohort studies, the identification criteria and diagnostic difficulties (5).

**TABLE 1.** Modified Oehlers’ classification of Dens Invaginatus

Type I	The invagination, which is enamel lined is of minor form.	No change
	It is confined within the crown of the tooth and does not extend beyond about the level of the external amelocemental junction.	
Type II	The enamel-lined invagination invades into the root but remains confined within it as a blind sac. There may, however, be a communication with the pulp. The invagination may or may not be grossly dilated.	IIA Invaginations extending up to the coronal third of the root canal IIB Invaginations extending up to the middle third of the root canal IIC Invagination extending up to the apical third of the root canal.
Type IIIA	Invagination extends through the root communicating laterally with the periodontal ligament via pseudo-foramen.	No change
Type IIIB	Invagination extends through the root communicating with the periodontal ligament via apical foramen. It usually does not involve the pulp.	

According to Ridell (6), the prevalence of type II DI is 79%, whereas types I and type III are 15% and 5% respectively. Although the aetiology of DI remains controversial and unclear, the depth of invagination dictates the morphology of the tooth from being an accentuated lingual pit to a severely malformed invagination. The presence of invagination makes the tooth more susceptible to carious lesions, pulpal infections and associated periodontal inflammation (2, 7-10). Due to the complex morphological variations, DI requires an early diagnosis although this rarely happens since it is mostly detected only when the patient presents with a large associated periapical lesion. Furthermore, the treatment of teeth affected by DI is fraught with difficulty.

This report aims to present different forms of DI type II, and propose subtype modification of Oehler’s classification that would aid in proper diagnosis and treatment plan of this anatomy.

## CASE PRESENTATION

This report follows the preferred reporting items for root and canal anatomy in the human dentition (PROUD 2020) (11). A total of 7 cases of DI constitutes this case series collected from 4 countries (Colombia, Hungary, India, Oman); with patient details and initial diagnosis given in Table 2. An informed consent was obtained from the patients who had reported for treatment. Though there were similarities in diagnosis and invagination patterns, the treatment protocol followed in each center differed. Some steps were common such as rubber dam application, modified access preparation under local anesthesia, exploration using DG16, troughing using ultrasonics, magnification using dental operating microscope, disinfection with copious irrigation with 5.25% sodium hypochlorite (NaOCl), final rinse with 17% (Ethylenediaminetetraacetic acid) EDTA, and calcium hydroxide (CH) intracanal medication (ICM). The manufacturer details of the products used have been mentioned in Table 3. The other details of treatment protocols are given below:

**TABLE 2.** Preliminary details of dens cases from different parts of the world

Age/sex	Country	Tooth no	Morphology	Type of dens*	Periapic-al lesion	Sinus tract	Investigation	CBCT	Diagnosis
27/M	India	22	Mesially tilted crown with a deep depression on palatal surface	II	+	+	No response	Yes-Small FOV	Pulp necrosis with asymptomatic apical periodontitis
25/F	India	22	Rotated peg shaped	II	-	+	No response	Yes-Small FOV	Pulp necrosis with laterally perforating inflammatory internal resorption
13/M	Hungary	12	Peg shaped	II	+	+	No response	Yes-Small FOV	Symptomatic apical periodontitis
13/M	Hungary	22	Peg shaped	II	+	+	No response	Yes-Small FOV	Symptomatic apical periodontitis
25/M	Colombia	12	Conical	IIIB	+	-	No response	Yes	Pulp necrosis with asymptomatic apical periodontitis
12/M	Colombia	12	Conical	IIIB	+	+	Normal	Yes	Normal pulp with chronic apical abscess
45/F	Oman	22	Barrel shaped	IIIB	+	+	No response	-	Pulp necrosis

*Type/classification of dens invaginatus based on Oehler's classification, CBCT: Cone beam computed tomography, FOV: Field of view

**TABLE 3.** Manufacturer details of the commercial products used

	Case 1	Case 2	Case 3	Case 4	Case 5	Case 6	Case 7
Instrumentation	Rotary- Protaper, Dentsply Sirona, Ballaigues, Switzerland	Rotary- Protaper, Dentsply Sirona, Ballaigues, Switzerland	Hand K-files	Hand K-files	Protaper Next, Dentsply Sirona	Reciproc R40, VDW, Munich, Germany	Protaper Next, Dentsply Sirona
Sodium hypochlorite	Prime Dental Products P Ltd, Thane, India	Ammdent, Chandigarh, India	Chlorax D 5.25%, Cerkamed, Poland	Chlorax D 5.25%, Cerkamed, Poland	Zonident, Proquident, Colombia	Zonident, Proquident, Colombia	Hyposol, Prevest Denpro Limited, Jammu, India
17% EDTA	Endoprep RC, Anabond Stedman Pharma Research (P) Ltd, Kanchipuram, India		Cerkamed, Poland	Cerkamed, Poland	EUFAR, Colombia	EUFAR, Colombia	Dolo Endogel, Prevest Denpro Limited, Jammu, India
Irrigation Device & Irrigant Activation		Endoactivator, Dentsply Sirona, Canada	Endovac, Kerr, Switzerland; NSX Varios 370, Satelec K20 tip	Endovac, Kerr, Switzerland; NSX Varios 370, Satelec K20 tip	XP-endo Finisher FKG, La Chaux de Fonds, Switzerland	XP-endo Finisher FKG, La Chaux de Fonds, Switzerland	
Intracanal medicament	Apexcal, IvoclaR Vivadent AG, Schaan, Liechtenstein	Avuecal, Dental Avenue, Palghat, India	Calcipast, Cerkamed, Stalowa Wola, Poland	Calcipast, Cerkamed, Stalowa Wola, Poland	Apexcal, IvoclaR Vivadent AG, Schaan, Liechtenstein	Apexcal, IvoclaR Vivadent AG, Schaan, Liechtenstein	Wellpex, Vericom, Korea
MTA	Angelus, Londrina PR, Brazil	Angelus, Londrina PR, Brazil			Angelus, Londrina PR, Brazil	Angelus, Londrina PR, Brazil	Angelus, Londrina PR, Brazil
Obturation/Gutta Percha	Thermoplasticised GP- Elements, Kerr Endodontics, Brea, CA, USA GP- Dentsply India Pvt. Ltd, Gurgaon, Haryana, India	Dentsply India Pvt. Ltd, Gurgaon, Haryana, India			Elements, Kerr Endodontics, Brea, CA, USA	Elements, Kerr Endodontics, Brea, CA, USA	
Sealer	AH Plus, Dentsply Sirona-DeTrey, Konstanz, Germany	AH Plus, Dentsply Sirona-DeTrey, Konstanz, Germany			Topseal sealer, Dentsply De Trey, Germany	Topseal sealer, Dentsply De Trey, Germany	
Composite	Te-Econom Plus, Ivoclar Vivadent, Liechtenstein	Ever-X Posterior and G-Aenial GC, Europe	GC Gradia-Direct A3 Alsin, IL, USA	Ever X Posterior, GC, Leuven, Europe	Tetric N-Ceram, Ivoclar Vivadent, Liechtenstein	Tetric N-Ceram, Ivoclar Vivadent, Liechtenstein	Fusion Ultra D/C, Prevest DenPro, Jammu, India
Glass Ionomer cement			Ketac Molar, 3M ESPE, Germany	Ketac Molar, 3M ESPE, Germany	Vitremer, 3M ESPE, MN, USA	Vitremer, 3M ESPE, MN, USA	
Temporary seal	Cavit G 3M ESPE, Seefeld, Germany	Cavit G 3M ESPE, Seefeld, Germany			Coltosol, Coltene Whaledent, OH, USA	Coltosol, Coltene Whaledent, OH, USA	Cavit G 3M ESPE, Seefeld, Germany

### Case 1: India (Fig. 1 a-e)

**Figure 1.**
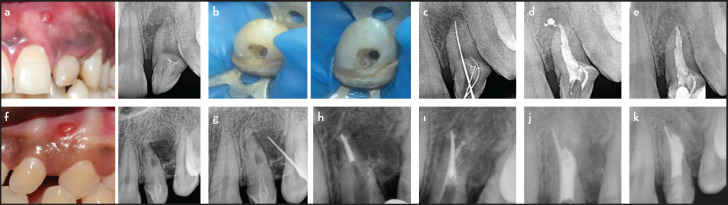
Type II DI cases: Case 1 depicts Type IIA root canal configuration (a) and its management (b-d) with 1 year follow up radiograph showing resorbed sealer (e); Case 2 depicts Type IIB root canal configuration (f, g) and its management using sectional obturation apical to the resorptive defect (h) and MTA obturation in remaining root canal space including resorptive defect (ı) post obturation and post- op radiograph (j, k)

Tooth #22 presented with a necrotic pulp, a periapical radiolucency and a sinus tract. Cone beam computed tomography (CBCT) scan (Myray iRYS, Cefla SC, Bologna, FSV 90 Kv, field of view (FOV) 8 x 5 cm, voxel size of 0.300 mm) was taken to analyse the morphological alteration. Three separate canals were identified and treated. The canals were negotiated by hand K-files (Mani, Tochigi, Japan) using balanced force technique and enlarged to size 30. Further chemo-mechanical preparation was carried out using rotary files in brushing motion to protaper F3 (Protaper, Dentsply Sirona, Ballaigues, Switzerland), with intermittent 5.25% NaOCl irrigation.

On the second visit, the sinus tract had healed. After thorough irrigation, the main canal was obturated by warm vertical compaction with Gutta Percha and AH Plus sealer. The distal invagination was packed and condensed with mineral trioxide aggregate (Grey MTA) using MTA carrier (GDC, Hoshiarpur, India) and Buchanan pluggers (Sybron Endo, Kerr Dental, Canada). The mesial invagination was filled by single cone obturation using AH Plus Sealer and GP (30/.06). The orifice was then sealed with resin composite.

A week later, a direct resin composite restoration was done for aesthetic management of the spacing between 21 and 22. The 1-year recall radiographic view showed complete resolution of the periapical radiolucency.

### Case 2: India (Fig. 1 f-k)

Tooth 22 showed evidence of an internal resorptive defect at the junction of the middle and apical thirds of the root, perforating laterally on the disto-palatal aspect with a lateral radiolucency which was evident on the CBCT images (New time cone beam 3D imaging, Cefla SC, Bologna, FSV 90 Kv, FOV 5x 5 cm, voxel size of 0.150 mm). Mechanical instrumentation was carried out up to F2 rotary file followed by circumferential hand filing with 40 K-file. Copious irrigation with activation using the EndoActivator (Dentsply Sirona, Canada) was done. Intracanal medicament was placed for two weeks, at which time the tooth was asymptomatic. Obturation was done using ProTaper F2 size master cone (Dentsply India Pvt. Ltd, Gurgaon, Haryana, India) using the sectional technique to the apical level of the resorptive defect. The rest of the canal was sealed with MTA. After 24 hours, the access cavity was sealed using short-fiber reinforced resin composite with a covering layer of hybrid resin composite restoration.

### Case 3: Hungary (Fig. 2 a-e)

**Figure 2.**
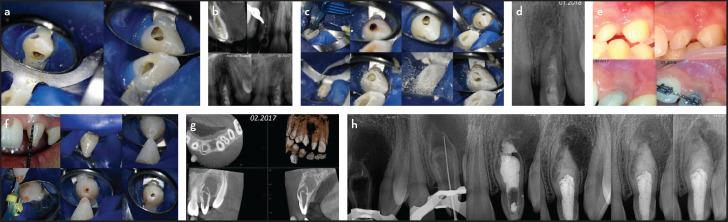
Type II DI cases: Case 3 depicts Type IIC root canal configuration (a) and its management using revascularization technic (b) followed by luting of glass fiber post (c) with 1 year follow up radiograph (d) and comparative preop and postop follow up photographs (e) Case 4 depicts Type IIC root canal configuration (f, g) and its management via placement of collagen and biodentine with post op and 3 years follow up radiograph (h)

Tooth #12 presented with one large root canal with a palatally located DI. The treatment was carried out in 3 sessions. Using CBCT (CS81003D, Carestream, Onex Corporation, Toronto, Ontario, Canada, FSV 90 Kv, FOV 5×5 cm, 0.075 mm) as a guide, the access cavity was initiated from the palatal for the DI with a secondary access from the buccal aspect to the apical region. The apex was scouted, working length determined and canal was instrumented using hand K-files up to size 70. Alternate activated irrigation was done using negative pressure irrigation and activated using a sonic device. The canal was dried using paper points and bleeding was induced using hand K- file reaching 1 mm beyond the WL. Biodentine (Septodont, Lancaster, PA, USA) was placed over the blood clot at the coronal third of the root canal and covered by glass-ionomer cement. During the same visit, a glass-fiber post (Nordic Glasix, Montreux, Switzerland) was luted with Rely-X Unicem (3M ESPE, USA) into the canal and the tooth was restored with a resin composite restoration. The 2-years follow up showed the tooth to be asymptomatic, and an increase in root length and dentine wall thickness was evidenced radiographically.

### Case 4: Hungary (Fig. 2 f-h)

The treatment of tooth 22 was carried out in 8 appointments. Since the DI was mainly communicating in the root, the tooth was preserved more coronally which was evident in the CBCT images (CS81003D, Carestream, Onex Corporation, Toronto, Ontario, Canada, FSV 90 Kv, FOV 5×5 cm, 0.075 mm). After mechanical instrumentation, double antibiotic paste containing ciprofloxacin and metronidazole was used as an intracanal medicament in the first session, while CH was used and replenished for the next 3 appointments. A membrane of collagen (Gelatemp, Coltene, Whaledent, USA) was placed before canal filling the apical portion of the root canal, upon which a layer of Biodentine (Septodont, Lancaster, PA, USA) and then glass ionomer was filled. A glass-fiber post (Nordic Glasix, Montreux, Switzerland) was then luted with Rely-X Unicem (3M ESPE, USA) and core build up was done using short fiber resin composite and hybrid resin composite. At 3-year follow up, the patient was asymptomatic and the recall radiograph shows bone healing.

### Case 5: Colombia (Fig. 3 a-e)

**Figure 3.**
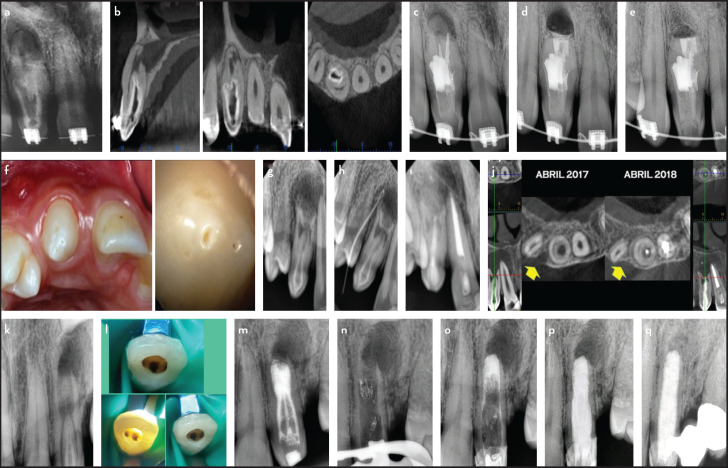
Type III DI cases and their management. 3 type III DI cases associated with large periapical lesions treated surgically (a-d), Non-surgical root canal treatment with warm vertical compaction (f-ı) and nonsurgical multi-appointment treatment with MTA apical plug and FRC post placement (k-p). Follow up after a year shows favorable healing (e, j, q)

Tooth 12 displayed DI with radiographic evidence of internal resorption demonstrated using CBCT (3D Accuitomo 170, Morita, USA, FSV 90 Kv, FOV 5×5 cm, 0.080 mm). The irregular canal and the invagination were instrumented using hand and rotary files, disinfecting intermittently with 5.25% NaOCl, activated using ultrasonics (Satelec Acteon, Merignac, France). Intracanal medicament was then placed for 2 weeks, after which obturation was done by warm vertical compaction with gutta percha and Topseal sealer. Endodontic microsurgery with root resection, retro-cavity preparation (E31D, NSK, Japan) and MTA filling was performed after 3 months to enhance the apical seal. The one-year recall shows complete bone healing.

### Case 6: Colombia (Fig. 3 f-j)

CBCT of tooth 12 displayed 3 invaginations evident on CBCT (3D Accuitomo 170, Morita, USA, FSV 90 Kv, FOV 5×5 cm, 0.080 mm); of these 2 were minor and were restored with resin composite. The larger canal was instrumented with a reciprocating system (R40, VDW, Munich, Germany) disinfected and Intracanal medicament was placed. Temporary seal of Coltosol (Coltene Whaledent, OH, USA) was given and the patient was recalled after 2 weeks. Teeth 11 and 21 were also necrotic and presenting apical radiolucencies.

On the second appointment, a sinus tract was still observed. Mechanical instrumentation was increased up to file size R50. The apical portion of the canal was instrumented manually up to ISO 110 K-file, disinfected and finally cleaned with XP-endo Finisher. MTA was packed in the apical third, confirmed radiographically and the patient was recalled a week later for final obturation with thermoplasticized GP. Coronal seal was ensured using glass ionomer cement followed by composite resin. Three months later, complete healing of soft tissues was observed. During this period endodontic treatments of teeth 11 and 21 was performed uneventfully. At 6 months, the patient remained asymptomatic and start of bone healing was observed radiographically in 12. The patient was reviewed again over time (1 year).

### Case 7: Oman (Fig. 3 k-q)

Tooth 22 with evidence of sinus tract had three separate entrances when accessed. After WL determination, the canals were instrumented up to size 25/.06, irrigated with 5.25% NaOCl and temporized with intracanal medicament and Cavit G. The patient, however, did not report back until after 6 months at which time, the tooth was found to be symptomatic with the sinus still present. The thin intercanal septa were removed and the now single canal was cleaned thoroughly using 5.25% NaOCl with passive ultrasonic activation, Intracanal medicament placed and access cavity sealed with glass ionomer cement. At the next visit, the patient was asymptomatic and the sinus had healed. After thorough cleaning of the canal to remove the Intracanal medicament completely, obturation was done with a 4mm apical plug of white MTA. A wet paper point was then placed over the MTA to allow it to set. At the recall visit, the paper point was removed and the rest of the canal was restored with 2 fiber posts and dual cure resin composite restoration. The 2-year recall radiographic view shows reduction of the periapical radiolucency with no clinical symptoms.

## DISCUSSION

During the morpho-differentiation phase of the tooth, an alteration in the proliferation within the enamel organ leads to the ingrowth of connective tissue through the dental papilla (12). This disrupts the ecto-mesenchymal signaling and causes in-folding of the enamel organ and cessation of further growth (13, 14). This developmental process may occur at different levels leading to variations in the morphological pattern of the dens, that will in turn dictate the treatment procedure.

According to literature, Ridell (6) reported that type I is the second most prevalent variant of DI. Since this invagination is superficial and partially involves dentine, it remains often undetected. A basic restorative procedure is the treatment of choice for the management of these cases.

Type II DI is the most commonly found morphological alteration (6). It is also noted that these remain undetected due to lack of symptoms in such teeth. De Smit (9) analyzed histologically 6 invaginated human maxillary incisors and reported that there was a structurally uniform layer of enamel present between dentine and the invaginated inner connective tissue layer that acts as protection, or as a portal for the entry for microorganisms, when it fails. Beynon (8) stated that the enamel lining at the base of the invagination is hypomineralized and irregularly structured. Similarly, Bloch-Zupan (15) studied the structural differences and compared the compositional variation between the outer and inner enamel. The authors demonstrated that the inner lining showed atypical and complex rod-shaped surface with honeycomb pattern and loss of perikymata. Chaniotis (16) also suggested that the invagination may cause a loss of blood supply. Hence both types II and III are generally not detected until a large periapical lesion develops over time as a result of pulp necrosis.

Oehlers’ classification is mainly formulated based on radiographic findings and it is useful in terms of clinical management. However, the case series presented in this report shows that management of these types could vary based on the different criteria, mainly for the extension of the DI to the coronal, middle or apical third of the root. Hence it is beneficial to undertake modifications of the existing classification based on the latest diagnostic tools and treatment techniques to enable the clinician to make an easier therapeutic decision.

The use of CBCT helps in not only identifying such invaginations, but also provides a three-dimensional reconstruction of the anatomical variations, defects and the associated periapical lesions. It also helps to alter the patterns of access design, cleaning and shaping protocols, and obturation methods, as described in our case series.

**Figure 4.**
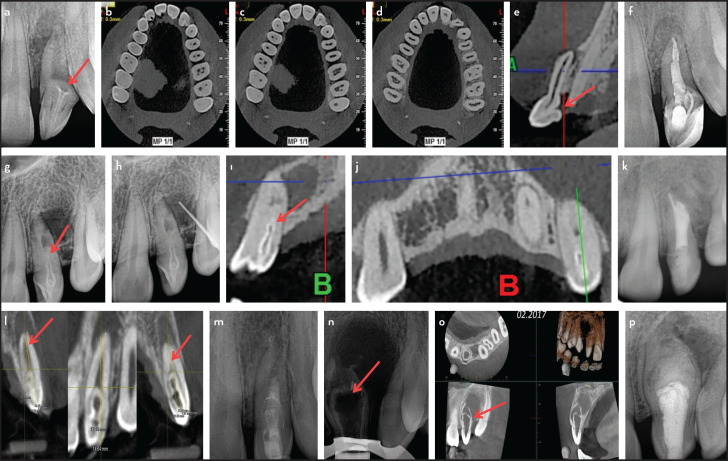
Different DI type II canal configurations. (a-f) Radiographs & CBCT images and post obturation radiograph depicting 3 canals in the coronal third (Type IIA). (g-k) Similarly type IIB with dens invading upto the middle third of the root canal. (l-p) Two type IIC canal configuration with dens extending to the apical thirds with 2 different treatment approach; revascularization (l, m) and Biodentine apical plug (n-p)

Of the 7 cases reported, 4 showed type II invaginations, while 3 revealed type III, where type 3 were addressed according to literature (Fig. 3). DI type II shows variations in the treatment approach depending on the extent of the invagination inside the root canal (Figs. 1, 2). The treatment approach differed where the invagination reached the apical part as compared to those where it did not. Also, where open apex was present with an apical diameter greater than ISO 110, a revascularization procedure was preferred. Hence, the authors suggest a sub-categorization of Oehlers type II based on the extent of the invagination, to be further classified as type IIA, IIB and IIC (Fig. 4) with invaginations extending up to the coronal, middle and apical thirds of the root canal respectively (Table 1). 

It was also interesting to note that type IIC resembled type IIIB and present with an open apex. Based on the presence or the absence of the invaginated lining and how early the tooth gets infected, the root dentine thickness of the main canal may vary. Thus, the authors recommend that such configurations presenting thin root canals walls (<1mm thick) be treated preferably using revascularization protocols to allow for root maturation (17, 18).

Due to the aberrant canal anatomy in DI Type III, achieving an intact apical seal is challenging. Therefore, a combined treatment approach of non-surgical root canal treatment followed by root-end surgery may become indicated. In the present case series, similar treatment protocol was followed in case 5 (Fig. 3 a-e).

Further, teeth with adequate root dentine thickness in both the DI types IIC and IIIB can be apically sealed with a tricalcium silicate-based material plug that would also facilitate bone regeneration with the use of an MTA apical barrier to promote osteogenesis at the infected site (19-21). The root may be also completely filled with tricalcium silicate in case intracanal space is not required to retain the coronal restoration. Also, the management of DI Type IIA (Fig. 1 a-e) and IIB (Fig. 1 f-k) extending to the coronal and middle thirds can be discernibly altered depending on the complexity and aberrant morphology which dictates the choice of root canal filling.

Although the treatment plan is case dependent, the treatment protocol needs to be addressed by the clinician after the evaluation of CBCT. Based on the present case series, it can be proposed that for DI type IIA and IIB, there can be effective conservation of tooth structure with thermoplasticized root canal filling into the main canal and MTA orthograde filling in the invagination. In type IIC, based on the root maturation (open or closed apex) the treatment can vary from revascularization for immature teeth and calcium silicate-based root end filling for effective apical seal followed by obturation and post endodontic management for closed apex.

## CONCLUSION

Based on clinical findings presented in this report, further sub-categorization of Oehlers DI type II into three subtypes representing the extension of the invagination into the coronal, middle and apical third is proposed. This enables the clinicians to decide on the root canal technique to be used. Further, the root canal filling protocol for type IIIB can be standardised based on the apical diameter and thickness of root dentine. 
